# Major League Baseball Marketing Strategies and Industry Promotion Approaches

**DOI:** 10.3389/fpsyg.2022.802732

**Published:** 2022-06-23

**Authors:** Meihong Chen, Fuzhi Su, Feng Tai

**Affiliations:** ^1^School of Physical Education, Dongguan Polytechnic, Dongguan, China; ^2^Department of Physical Education, Dongguan University of Technology, Dongguan, China; ^3^College of Athletics, Liaoning Normal University, Dalian, China

**Keywords:** promotional strategies, marketing strategies, sport of baseball, sports industry, baseball analytics

## Abstract

The sport of baseball is one of the chief pillars of the American sports industry. Major League Baseball (MLB) is the oldest professional sports league in the United States. It has long since formulated comprehensive marketing strategies and global industry promotion approaches that have proved exceptionally successful. Accordingly, the study of MLB's marketing strategies and industry promotion approaches will be crucial for the development of baseball in China and the establishment of an industrial chain. This study employed the literature consultation method, the comparative analysis method, and the inductive method to analyze MLB's localized marketing strategies and development trends in China and obtained the following insights concerning MLB's promotion and industry development efforts in China: (1) MLB has used a “family sport” concept to promote baseball culture and employed a project culture approach to promoting the universal spread of the sport of baseball; (2) MLB has sought to join baseball with school sports as a means of developing baseball talent; (3) MLB has promoted its brand, established a baseball industry chain, and engaged in comprehensive market cultivation; and (4) MLB has strengthened baseball infrastructure and encouraged baseball's rapid development.

## Introduction

In 2016, the International Olympic Committee announced that baseball would be a formal competition item at the 2020 Tokyo Olympics. Returning as an Olympic sport after a 12-year hiatus, baseball was sure to embark on a period of increased global popularity. At the same time, baseball's upbeat prospects would certainly lend a certain degree of impetus to baseball's spread, promotion, and industrial development in China. The sports industry of China is currently at an early stage of development. The number of people participating in sports has been steadily increasing. A growing number of resources have been directed toward sports, and the sports market has been expanding, and the large-scale commercialization and industrialization of sports appear imminent. However, a comprehensive market industry ecosystem will require the entry of more types of sports with relatively mature professionalization, industrialization, and market development. As the world's leader in the development of professional baseball, Major League Baseball (MLB) possesses a mature operating model and advanced industry development concepts. It has tremendous influence in the United States and other countries (such as Korea and Japan). As a consequence, the study of MLB's marketing strategies and market development approaches, as well as MLB's promotion plans and pathway choices in China, will have a great reference value and practical guidance applications in connection with the development of the sport of baseball and the baseball industry in China (Armstrong and Kotler, 2011; All-China Sports Federation, 2017).

## Literature Review

### Sports Marketing

The sports marketing environment can be identified as theme-based, product-based, alignment-based, and sports-based strategies.

- Theme-based strategies: Theme-based strategies can be defined as the use of traditional marketing strategies that incorporate a sports theme into the marketing program for non-sports products. The marketer might opt to use a sports-related copy platform or advertise products in sports-related media to effectively reach customers (Fullerton and Merz, 2008).
- Product-based strategies: Efforts to market sports products using traditional marketing strategies when the marketer has no official relationship with the sports entity being used in its marketing efforts are classified as product-based strategies. These strategies may or may not involve a sports theme beyond the product offering (Fullerton and Merz, 2008).
- Alignment-based strategies: Many marketers of non-sports products officially align themselves with sports properties *via* one or more of the four forms of sponsorship (traditional sponsorships, venue naming rights, endorsements, and licensing agreements). The nature of this sponsorship-based relationship reflects a higher level of integration of sports within the sports marketing environment (Zhao et al., 2010; Zhang, 2015).
- Sports-based strategies: This domain is characterized by official sponsors of a sports property who are selling other sports products (Zhang and Zhang, 2009; Ye et al., 2019). Because of the role of sports in both the product and integration dimensions, this domain may reflect the greatest reliance on sports-oriented initiatives. For example, Adidas sells sporting goods, and it uses advertising that complements its traditional sponsorship of FIFA and the World Cup of Soccer. This consistency produces the synergy characteristic of the sports-based domain (Fullerton and Merz, 2008).

### MLB Market

“Our plan is to put an emphasis on promoting our players and engaging a more diverse audience,” said Barbara McHugh, MLB's senior vice president for marketing. “Baseball should put more money into promoting these guys, any way possible, across all avenues, social media, TV, in person; baseball faces limitations in other sports. The game doesn't really lend itself to a lot of flash,” he said. “Posing and watching your home run go over the fence, and the long, slow home run trot, baseball tends to legislate against it (Yang et al., 2012; Ye et al., 2021). They should loosen things up a little more and let these guys show more personality. MLB Major League Baseball Promotion & Advertising Strategy: Major League Baseball (MLB) uses extensive promotion using TVCs, merchandising, online ads, media coverage, etc. (Badi, 2015). Most stadiums have their own ways of promoting the matches that they are going to host. If the match is between two rival teams, the amount of effort required by the promoters is less. Promotions are sometimes done through hoardings, banners, billboards, TV commercials, print media, and radio stations. Since this is a service marketing brand, here are the other three Ps to make it the 7Ps marketing mix of Major League Baseball (MLB). Major elements: People: Process: Physical Evidence (Dibb et al., 2005; Cho et al., 2012).

## MLB'S Marketing Strategies

According to official data, over 70 million people attended MLB games in the United States during 2016, and the value of MLB products, including tickets, licensed merchandise, retail items, and media broadcasts, exceeded USD$10 billion, placing MLB at the top of the world's professional sports leagues (Liu et al., 2005, 2014). MLB has relied on its unique industry development strategies and marketing system. Its distinctive anti-trust immunity system has enabled it to lay a strong foundation for its continued robust growth. In addition, MLB has progressively devised a sports-centered baseball industry chain and baseball industry marketing strategies in the case of its steady growth. In his book *Sports Marketing: A Strategic Perspective*, Matthew D. Shank asserts that sports marketing refers to “the specific application of the principles of marketing to sports products and the reliance on sports to market non-sports products; this is sports marketing.” Based on the 4P marketing principles and Matthew Shank's definition of sports marketing, this study divides sports marketing into product strategies, price strategies, channel strategies, and publicity strategies and relies on these four dimensions to investigate and analyze MLB's marketing strategies (Matthew et al., 2002; Lin et al., 2020).

### Creating Branded Games, Enhancing Product Marketing Strategies

Sporting events are one of the core elements of the sports industry's development (Huang et al., 2019; Fu et al., 2022). MLB is one of the four major professional sports leagues in North America and currently has 30 major league teams; Minor League Baseball (MLB) is positioned under the major league teams. Each major league team has affiliated teams at, at least, six levels—this is known as a farm system. Each major league team's farm teams compete against each other in their respective leagues, and there are six lower-level baseball leagues, which provide an all-inclusive league system. MLB's competition system comprises regular games, all-star games, and playoff games (Sun, 2015). Traditional games are typically conducted from April to October each year, and every major league team takes part in 162 regular games during each season. Thanks to the teams' long schedules and many games, MLB fans have many viewing options. The essence of a sporting event lies in the quality of the play and the spectators' experience and impression (Li, 2001; IEG, 2017). Accordingly, MLB has consistently sought to promote a fan-orientated stadium development concept; as a result, MLB baseball stadiums are not just places where fans can watch games but offer multiple functions and provide fans with a wide range of services and experiences. In summary, MLB ballparks provide spectators with an entertaining atmosphere. Apart from sporting events, MLB's franchises are another essential part of its product (Liang, 2015; Pan, 2018). MLB's team uniforms are currently provided by Nike and Majestic, and products bearing the MLB mark or the logos of individual teams, namely, sports clothing, baseball shoes, hats, and backpacks, are sold at specialty stores (Goss, 2009; Mihai, 2013). The most distinctive product at MLB franchise stores consists of baseball cards, which are collectors' items and hold a certain amount of value and the potential for increased value (Chinese Baseball Association, 2015). According to international practice, all baseball cards are printed and issued in limited editions, which ensures that each baseball card possesses great commemorative significance and value (Hua'ao, 2014; Huang et al., 2017). Income from the sale of licensed MLB products totals over $3 billion each year (Drayer and Rascher, 2013; Huang et al., 2015).

Apart from routine event handling and franchise management, each MLB team is actively involved in the development of auxiliary projects aimed at increasing their business income. The most common of these are the service facilities the teams have built around their stadiums, which include office buildings, residential buildings, commercial pedestrian streets, places of entertainment, restaurants, and other retail shops. These projects help realize integrated sports business models (Chen et al., 2020b, 2021).

### Strategies for Boosting Market Share and Optimizing Product Pricing Strategies

According to the data, the ticket prices for major league baseball games in the United States are significantly lower than those for games of the three other major sports leagues [the National Football League, National Basketball Association, and Professional Golfers' Association (PGA)]. This is because MLB's product pricing has consistently tended to be consumer-friendly, which reveals MLB's efforts to capture a large market share through massive sales with a low-profit margin (Chen et al., 2015, 2019). Furthermore, MLB's establishment of infrastructures such as large stadiums and commercial centers has provided spectators with year-round multiple-venue, multi-level, and multiple-grade baseball games, which can satisfy the needs of different spectators. This approach has steadily boosted MLB's demographic participation and popularity, maintained its market share, and created industry value and a steadily increasing overall income (Cheng, 2012; Chen et al., 2020a).

Apart from its reasonable product pricing, MLB has also exhibited innovation in its ticket sale formats. For instance, after dividing fans into categories, MLB established special seats and discount tickets for the disabled and has initiated “family tickets” and “group tickets” for families and groups. These measures have increased the empty seat utilization rate and met the needs of various groups. In addition, within the major league framework, MLB teams possess a high degree of autonomy in setting ticket prices. Each team can flexibly set prices according to its own specific situation. For instance, the Chicago Cubs—winners of the 2016 World Series—announced a ticket price increase plan calling for an average price increase of 19.5% for the season after winning this championship for the first time in the team's history. While consolidating conventional ticket sale income, MLB addressed the impact of new media on fans' live baseball viewing and the changes in younger fans' viewing habits by taking advantage of its new media platforms to promote diversified ticket sale strategies, which have included reducing the price of mlb.tv, providing more media products, and introducing 2020 online broadcast packages for individual teams.

Regarding the use of smartphone apps as a ticket sales channel, in 2015, the Atlanta Braves, Chicago White Sox, and Oakland Athletics led the way by releasing a “Ballpark Pass” as an official MLB app, which soon took the rest of the league by storm. At present, 22 teams currently use the Ballpark Pass app, which lets spectators who pay a monthly fee view all home-field games. According to MLB's media statistics, as of June 2017, a total of over 500,000 tickets were sold *via* Ballpark Pass, which was 2.5 times the amount of Ballpark Pass ticket sales during the entire 2016 season.

Thanks to its correct pricing strategies, the total number of MLB in-stadium spectators has exceeded 70 million over the last few years, placing MLB at the top of the global league sport attendance rankings. The New York Yankees' 2014 ticket sales revenue surpassed USD$300 million, and USD$3 billion of total MLB income exceeding USD$10 million in 2016 was attributable to ticket sales.

### Distribution Strategies for MLB Brand Products

Sporting goods superstores and stadiums are the most common distribution channels for sports-related products, and various types of chain sporting goods stores and sporting goods superstores can be found all over the United States. The uniforms for the MLB teams are currently provided by Nike (contract signed in 2009) and Majestic (contract signed in 2005). Both Nike and Majestic have very strong retail systems in the United States and their online/offline retail channels all over the country. While the most conventional distribution channel connected with sporting events is the sale of products to spectators watching games, this conventional, uniform distribution channel has long been unable to satisfy consumer demand, and MLB has found that media distribution approaches provide an excellent supplement to its conventional distribution channels. With regards to media cooperation, MLB has signed game broadcast contracts with TV media companies such as ESPN, Fox, and Turner Broadcasting, which allows consumers constrained by time or geographical factors to watch games at any time or place. In the e-commerce domain, MLB established a cooperative relationship with the new US vertically-integrated e-commerce sporting goods platform Fanatics in 2003, and Fanatics has provided MLB with an online sales channel. Fanatics relies on its high-efficiency distribution method to ensure that the newest MLB products are presented to consumers *via* Fanatics' e-commerce platform within the space of a few hours, which greatly increases the distance between consumers and MLB products. Apart from cooperation with other companies, MLB established the Major League Baseball Advanced Media Company (MLBAM) in June 2000 to bear exclusive responsibility for disseminating MLB information online. MLBAM helps fans understand MLB while also burnishing MLB's reputation and influence. MLBAM currently operates the websites MLB.com, MiLB.com, MLB.TV, and Gameday Audio. These websites also provide links to all the league's teams and serve as additional display windows, enhancing team publicity and product sales.

Among the various event broadcast media, Fanatics' powerful online sales network, interlinked sporting goods stores, and mature stadium operating model enable it to provide MLB with a highly-effective product distribution network. Both online and offline, MLB can directly and effectively target consumers and can not only present products to consumers *via* these channels but also take advantage of these channels to obtain consumer feedback, allowing it to provide consumers with even better products and services.

### MLB's Product Publicity Strategies

Publicity strategies such as the use of corporate sponsors, game broadcasts, and new media marketing have played enormously important roles in MLB's development. In the case of sponsors, during the 2017 season, MLB's sponsorship income totaled more than USD$892 million, and the three leading sponsor categories consisted of retailers, car manufacturers and fast food industries, and insurance and food products. MLB corporate sponsors are typically in industries that have close connections with people's everyday lives, which intangibly increases MLB's influence on the American public. As for game broadcasts, MLB signed an 8-year broadcasting contract worth USD$5.6 billion with ESPN in 2012, and MLB also jointly signed an 8-year contract expiring in 2021 with Fox and Turner Sports. MLB signed a cooperative broadcast agreement with Facebook in May 2017, allowing American fans to watch one direct-broadcast big-league game each week *via* the Facebook Live platform. At the same time, each MLB team has also negotiated its own local broadcast agreements. These broadcast agreements not only bring in vast amounts of income for the league but also provide an excellent pathway for further enhancing the league's publicity. For mobile users, MLBAM has provided MLB with strong Internet operating capabilities and technical support, and the official “At Bat” MLB app, developed and operated by MLBAM, holds the first-place rank among American iTunes sports app downloads. At least 10 relevant apps are currently available on MLB's official website.

The immense influence of MLB on the American public is inseparable from the breadth and effectiveness of the league's publicity strategies. MLB has taken baseball culture as a point of entry, which has made MLB a popular street fashion and sports brand in the United States. Apart from baseball's original cultural status as a popular outdoor sport, a classic, nostalgic sport, and a heroic sport, the star effect created by MLB has also strengthened the league's appeal in the public's eye. MLB has perfectly fused competitive sport, leisure recreation, and social interaction in one activity, which is something that other sports have not been able to accomplish. This culture has permeated every corner of American society, and taking the whole family to watch a baseball game on the weekend before returning home to eat together is a traditional activity for American households.

## MLB'S Promotional Strategies in the Market

Since 2002, MLB has actively targeted the Chinese sports market and has been in contact with the Chinese Baseball Association since 2003. MLB has sponsored the Chinese national baseball team's equipment, provided instruction to coaches, and guided research. Subsequently, in keeping with its greater assistance and contact, MLB has not only provided free training services for Chinese athletes, umpires, and coaches but has also shown the association how it can promote and popularize the sport of baseball among the younger generations and how it can successfully adopt MLB's baseball culture, concepts, products, and games in China. MLB has worked tirelessly to develop baseball in China, which has promoted MLB and its industry. On January 6, 2016, MLB announced its formal entry into the Chinese market in Las Vegas.

### Promotional Concept: A Sport for the Whole Family

“Mom can be the pitcher, the son can be the batter, and dad can be the catcher: This is the way the whole family can participate together.” This is MLB's key slogan and concept as it strives to promote baseball in the Chinese sports market. Beyond being a popular sport, baseball is also a traditional family and community activity in the US. For instance, families get together to watch baseball games, and hitting baseballs or playing catch at ball fields across America is a way that fathers teach their children baseball skills; it is also a traditional American household activity. Baseball culture has helped bind America and has maintained baseball's long-term status as a leading mainstream sport. When formally entering the Chinese market, MLB's CEO emphasized that “Baseball is a sport that takes the whole family as a unit.” Furthermore, according to Gao Fei, President of LeSports: “Scoring occurs in baseball games when a runner ‘returns home' (to home plate), which happens to be completely in keeping with the traditional concept of family in China. However, it is very rare for Chinese family culture to involve this kind of sport, and it is much more common for parents to take their children out to eat or on a trip. Most parents are not in the habit of playing sports with their children. As a consequence, MLB has been consistently relying on the educational system and the community to encourage more young people to participate in baseball. LeSports is also encouraging a growing number of families and young people to participate and enjoy this extraordinary sport.” Internal factors are often the best promoters of development, and MLB is relying on its distinctive cultural concept of baseball as a “family support” to promote baseball in China, and this approach is in keeping with China's traditional cultural concepts.

### MLB Strategies for the Marketing and Sale of Branded Merchandise

When MLB entered the Chinese market in 2007, it first began promoting its clothing products and established ~300 authorized brand product specialty stores in 10 of China's first- and second-tier cities. Although the merchandise at these stores was priced relatively high for sports products, the products' novel styles and designs enabled the stores to attract millions of fans. In addition, as MLB's official baseball cap manufacturer and distributor, New Era has established physical stores and an online flagship store in China and, to date, has launched 15 stores in nine Chinese cities, including Shanghai, Shenzhen, Chengdu, Suzhou, and Nanjing. New Era has further established cooperative agreements with such Chinese fashion stores as FOSS and Folder, providing the display of New Era's merchandise in those companies' offline stores. Apart from promoting existing products, New Era also established a strategic alliance with the Giant Interactive Group in November 2016 to enable New Era's products to formally enter the E-sports peripheral merchandise market in China. It can be seen that MLB has been accelerating its entry into the Chinese clothing market in recent years, and the number of specialty stores established by its authorized clothing distributors in first- and second-tier Chinese cities has been growing exponentially. At the same time, New Era has also accelerated its deployment in the Chinese market this year. Thanks to its reliance on authorization, the establishment of directly operated specialty stores, and cooperation with other brands, MLB has succeeded in marketing its peripheral merchandise both online and offline in China; it has diverse retail channels at its disposal, and its sales network is approaching completion.

### Construction of a Youth Baseball Development System, Establishment of a Basis for the Industry's Development

MLB has been promoting itself as a core sports product in China. By promoting the MLB brand in China and simultaneously burnishing its international brand image, MLB has contributed to American baseball's global development and influence. In 2007, MLB initiated the “MLB Play Ball! Nationwide Youth Baseball Development Plan” in China, embarking on the development of Chinese youth baseball. The purpose of this initiative is to broadly improve the health and fitness standards of elementary and high school students throughout the country in keeping with the “Sunshine Sports” development directive jointly issued by the Ministry of Education, the General Administration of Sport, and the Communist Youth League Central Committee. The MLB's plan consists of two parts: a school baseball popularization plan and the establishment of youth baseball leagues: (1) The school baseball popularization plan is mainly aimed at the vast number of elementary and high school students in China, and it seeks to teach them baseball knowledge and provide them with basic baseball training in both physical education classes and extracurricular activities. The plan is to currently provide resources, including skills training, equipment, and instructors, to in-school students and physical education teachers at more than 180 elementary schools and high schools nationwide and to conduct coach-assissted training camps and rely on physical education classes to provide baseball instruction broadly to elementary and high school students throughout China. Close to 500,000 in-school students in China are receiving basic baseball training, and this effort provides a vast pool of potential baseball talent. The “College Club” plan, initiated by MLB in 2009, has established over 70 baseball clubs at more than 70 universities, providing students opportunities for autonomous athletic activity, and has also held baseball talks and team training activities for individual clubs, as well as providing the clubs with the equipment and support they need. MLB has also organized city-wide university team games in Beijing and Shanghai and is expanding its efforts into the southwestern and central areas of China. (2) Youth baseball leagues: The “MLB Play Ball!,” the youth baseball league, was formally established in 2008 and is currently the sole nationwide Chinese youth baseball league. This league has thus far held 2,168 games in six cities. The “MLB Junior Play Ball!” youth baseball league is an extension of the MLB Play Ball! League and it targets students who have only recently encountered baseball; most players participating in games are in fourth grade or below. The MLB Junior Play Ball! league is an all-new part and an important element of MLB's plan to promote youth baseball in China. MLB Junior Play Ball! not only holds formal games but also conducts distinctive parent and child games, promoting family interaction. This has highlighted the family culture that underlies the sport of baseball and gives kids and their parents a chance to enjoy the fun of baseball. During the 2017 season, MLB Play Ball! conducted games under a comprehensive promotion/demotion system and seamlessly integrated with MLB Junior Play Ball! The playing periods of the leagues lasted an entire year, and they strove to attain a new, higher operating standard and level of competition. During the 2017 season, MLB Play Ball! league conducted city-wide competitions from March to May in the four major areas of Beijing, Shanghai, Guangdong (Guangzhou and Shenzhen), and Chengdu. The MLB Junior Play Ball! league held competitions from April to June in the eight areas of Beijing, Shanghai, Guangzhou, Shenzhen, Chengdu, Jinan, Shijiazhuang, and Changsha.

Furthermore, China's first baseball development center was established in Wuxi in 2009, followed by the establishment of a second baseball development center in Changzhou in 2011 and the third center in Nanjing in 2014. These three baseball development centers are separated by ~2 h of travel time by high-speed rail and provide an excellent means of promoting everyday contact and instructional competition between baseball learners in these three areas and demonstrate MLB's developmental model in the Jiangsu region. At the same time, MLB has also established ballparks in over 20 large and medium-sized cities nationwide. These ballparks have induced more than three million young people to come into direct contact with the sport of baseball. In summary, apart from holding training camps for coaches and umpires and establishing baseball leagues for university, high school, and elementary school students, MLB has also established baseball development centers for the purpose of popularizing and promoting baseball while also cultivating large numbers of baseball enthusiasts and business operators. At the same time, these measures are training specialists for China's baseball market and further creating an event-centric baseball industry chain in the Chinese sports market.

In contrast to the NBA, MLB has been involved in the Chinese sports market for a relatively short time, and the public basis for the expansion of baseball is still relatively weak. Accordingly, long-term, intensive planning and action will be needed to cultivate a fanbase and develop the baseball market. Aware of this reality, MLB has directed its chief efforts toward creating a youth baseball population since entering the Chinese market and has relied on MLB Play Ball!, MLB College Club and its baseball development centers as a “starter” for the promotion of youth baseball. As key elements of MLB's chief strategy for the promotion of baseball in China, MLB Play Ball!, MLB College Club, and MLB baseball development centers focus on clearly defined demographic segments and have clear-cut goals. While promoting and popularizing the sport of baseball among young people, MLB has been involved in training an outstanding pool of baseball talent for China.

### Making Use of Media Publicity to Implement MLB's Brand Strategy

MLB has been paying close attention to the rapid development of new media platforms in China and embarked on the establishment of the MLB media system in the wake of 2010. MLB's media utilization goal is to boost MLB's brand image, name recognition, and public awareness. Currently, MLB's TV team in China has secured rebroadcast rights and coverage and broadcasts major MLB games, including opening games, all-star games, and World Series games, each season, while also producing the “Baseball Weekly” program. In 2011, MLB began cooperating with *Sports Weekly*, which established an MLB column, and *Sports Weekly* has become a vital print media partner for MLB in China. In 2014, MLB joined forces with LeSports to produce “MLB's My Player,” a reality show. The two partners conduct a joint promotion in an MLB ballpark in China each year, with concurrent online promotions of the two activities. In order to quickly expand its influence, on April 3, 2015, MLB signed pop singer Jane Zhang as the first MLB Ambassador for China, and it has also made Allen Lin and Liu Jianhong MLB promotional ambassadors. Cementing the relationship with new media, MLB made LeSports its official strategic partner for China in 2016. The two partners have achieved a full-scale cooperative relationship in the areas of online services and offline promotion. LeSports will establish online communities for each MLB team, which will bring together China's baseball community. In terms of content, LeSports will rely on cell phones, PCs, and apps and take advantage of its existing direct broadcast content foundation to provide direct broadcasts of a majority of MLB games on all platforms; it will also broadcast domestic events, including MLB's collegiate championships, Shanghai university student baseball championships, and the MLB Play Ball! League championships. China Education Television is one of China's two TV networks with nationwide coverage (the other being CCTV) and covers 971 million of China's citizens. It is also the only domestic TV network reaching more than 400,000 K-12 schools and over 2,000 universities and colleges. It has been reported that since China Education Television established a strategic alliance with MLB in 2017, the two partners have jointly produced nine TV programs, allowing countless young people throughout China to watch high-quality baseball events worldwide on China Education TV.

Taking advantage of conventional media, MLB has signed a number of influential entertainers and sports announcers to serve as promotional ambassadors for MLB in China. MLB has relied on the influence of these figures to raise awareness of MLB in China. With the rise of new media in China, many video media and gateway website operators, including Tencent, Sohu, and LeSports, have emerged, and new media haw occupied an increasingly prominent role in China's media ecology. In particular, new media's cultural orientation and event broadcasting ability are especially noteworthy. New media has become an important link in MLB's handling of public relations. With regards to its publicity system, MLB's everyday media publicity system chiefly relies on the central media while taking advantage of local media in a supplementary role and makes primary use of online media while making supplemental use of TV and print media. In summary, the media system that MLB has preliminarily established in China is steadily enhancing MLB's brand awareness and influence in China.

## Conclusion

Even though baseball has been expanding in China for over 60 years, it has never truly achieved significant growth momentum. It is not difficult to identify the reasons behind this, which include the persistent “old problems” of insufficient popularity, poor public awareness, and a lack of infrastructure, all of which require immediate attention. However, we are currently living in a time when China's sports industry has begun to develop, market demand is increasing, and industrialized, market-oriented, professional sports are steadily entering the market. As a result of the impetus provided by changing times and the opportunities for development that they have provided, MLB has identified the Chinese market as an important piece of territory in its campaign to develop baseball globally. MLB is therefore relying on China's vast sporting population and huge market potential to vigorously promote Major League Baseball and its associated industry. In recent years, MLB has employed the “family sport” concept to find common ground with China's traditional culture and concept of the family and use this conceptual commonality to promote baseball among young people and families. As a result, MLB has gradually established a distinctive baseball culture in China and has greatly increased the number of people who play baseball. At the same time, MLB has helped support baseball leagues of all levels and types and has relied on these leagues as a basis for the creation of a baseball industry chain. These approaches have consistently been a marketing method for MLB in China. Finally, as a consequence, the study of MLB's industry marketing strategies and industry promotion pathways will have important significance and value for the global development of sports enterprises in China and the establishment of an industrial chain.

## Author's Note

The research results, it show that in the original data set and the filtered data set, the prediction effect of limit gradient boosting (XGB) is the best, and the average MAPE index is only about 2-3, which is better than other methods. It can be seen that the XGB method has a better fit to the regression problem and high interpretability, and has the ability to accurately predict and evaluate the NBA score prediction. In future work, it is expected that this method and model can be integrated with the latest NBA season data to provide analysis and prediction results for NBA teams, coaches, management units, and related stakeholders, and provide a comparative analysis of the team to the players. The results can support the team's consideration of player management decisions making.

## Data Availability Statement

The original contributions presented in the study are included in the article/supplementary material, further inquiries can be directed to the corresponding author/s.

## Author Contributions

Material preparation, data collection, and analysis re performed by MC. The first draft of the manuscript was written by FS. Revised manuscript improved by FT. All authors contributed to the study conception and design, read, and approved the final manuscript.

## Conflict of Interest

The authors declare that the research was conducted in the absence of any commercial or financial relationships that could be construed as a potential conflict of interest.

## Publisher's Note

All claims expressed in this article are solely those of the authors and do not necessarily represent those of their affiliated organizations, or those of the publisher, the editors and the reviewers. Any product that may be evaluated in this article, or claim that may be made by its manufacturer, is not guaranteed or endorsed by the publisher.
